# Postoperativ sich entwickelnde ulzeröse Hautläsionen mit assoziierter Colitis ulcerosa bei einem Patienten mit Z. n. femorokruraler (P3) Bypasseinlage wegen peripherer arterieller Durchblutungsstörung

**DOI:** 10.1007/s00104-024-02190-y

**Published:** 2024-11-08

**Authors:** U. Barth, M. Eltokhy, F. Meyer, D. Langer, Z. Halloul

**Affiliations:** 1 Arbeitsbereich Gefäßchirurgie, Klinik für Allgemein‑, Viszeral‑, Gefäß- und Transplantationschirurgie, Universitätsklinikum Magdeburg A. ö. R., Leipziger Str. 44, 39120 Magdeburg, Deutschland; 2 Klinik für Allgemein‑, Viszeral‑, Gefäß- und Transplantationschirurgie, Universitätsklinikum Magdeburg A. ö. R., Magdeburg, Deutschland; 3 Institut für Pathologie, Klinikum Magdeburg gemeinnützige GmbH, Magdeburg, Deutschland

## Anamnese

Ein 80-jähriger Patient wurde mit einer inkompletten Ischämie des rechten Beines notfallmäßig nach kompletter perkutaner transluminaler Angioplastie (PTA) und Implantation von 3 selbstexpandierenden Stents (Visi Pro, Medtronic, Minneapolis/MN, USA) bei langstreckigem Verschluss der A. femoralis superficialis rechts, die am Vortag stattgefunden hatte, aufgenommen. Der Patient klagte über starke Ruheschmerzen und eine kühle untere rechte Extremität bei erhaltener Sensibilität. Daraufhin wurde die Revaskularisation durch Anlage eines femoroinfragenualen Bypasses mittels beringter Gore Propaten^®^-Prothese (W.L. GORE, Putzbrunn, Deutschland) mit einem Durchmesser von 7 mm durchgeführt. Nach einer kurzen Überwachungsphase auf einer Intermediate-Care-Station konnte der Patient auf die Normalstation verlegt werden. Im weiteren Verlauf entwickelte der Patient blutig tingierte Stuhlgänge. Daraufhin erfolgte eine Koloskopie. Sowohl das klinische und endoskopische Bild als auch die Histologie bestätigten die Diagnose einer schweren Colitis ulcerosa. Die Behandlung erfolgte oral und rektal mit Mesalazin (Salofalk, Dr. Falk Pharma, Freiburg, Deutschland), worunter sich das Beschwerdebild deutlich besserte. Des Weiteren entwickelten sich im Bereich des rechten Unterschenkels in Höhe des medialen Malleolus einschmelzende Ulzerationen, später im Verlauf auch am linken Unterschenkel.

## Klinischer Befund

Initial zeigte sich am Malleolus medialis rechts ein ca. 3 × 3 cm großes gerötetes und schmerzhaftes Areal mit einer ca. 5 mm großen gelblichen Pustel mit danebenliegenden kleineren Pusteln, zum Teil nässend und mazeriert. Im kurzfristigen Verlauf kam es zur Progredienz des pustulären Areals mit Auflösung der Dermis und Mazerationsbildung.

## Diagnostik

### Labor

In der Entwicklungsphase der pustulären Veränderung zeigten sich die Leukozyten im Normbereich. Es kam zu einem moderaten Anstieg des CRP von 67,3 mg/l auf 74,6 mg/l. Der Hb-Wert war bei 4,9 mmol/l stabil auf niedrigem Niveau. Die entnommenen mikrobiologischen Abstriche aus den pustulären Veränderungen waren steril.

### Bildgebung

**Abb. 1:**
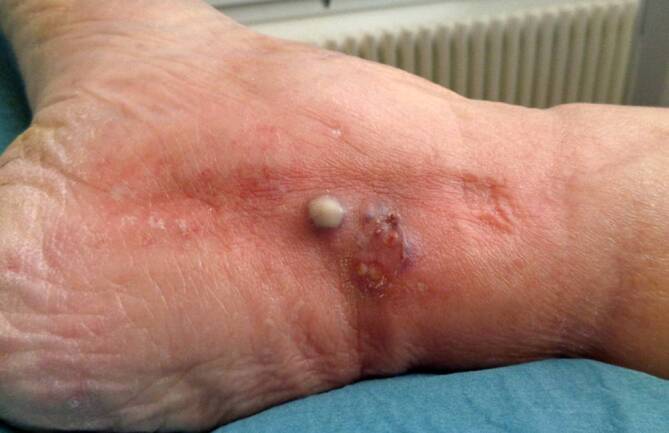
Initialbefund am medialen Malleolus der gefäßchirurgisch sanierten Extremität – klinische Fotodokumentation des Lokalbefundes der medialen Malleolarregion (rechte untere Extremität) im stationären Behandlungsverlauf (Quelle: klinischer Bilderfundus – Arbeitsbereich Gefäßchirurgie; Klinik für Allgemein‑, Viszeral‑, Gefäß- und Transplantationschirurgie)

**Abb. 2:**
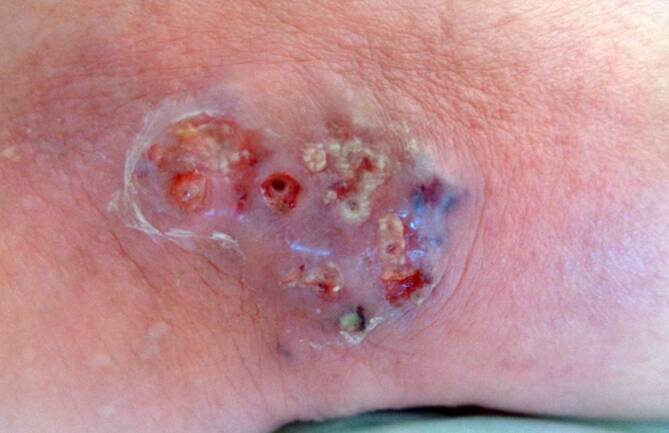
Verlaufsstadium des Lokalbefundes – klinische Fotodokumentation des Lokalbefundes der medialen Malleolarregion (rechte untere Extremität) im stationären Behandlungsverlauf (Quelle: klinischer Bilderfundus – Arbeitsbereich Gefäßchirurgie; Klinik für Allgemein‑, Viszeral‑, Gefäß- und Transplantationschirurgie)

Die Fotodokumentation spiegelt den progredienten Verlauf der pustulären Effloreszenz wider. Hier ist eine dynamische Größenzunahme zu erkennen. Abb. 1 zeigt den Initialbefund. Im weiteren Verlauf kam es zu einer zirkulären Ausbreitung der Effloreszenz (Abb. 2), die unter dem Verdacht einer Abszessbildung bei frisch implantierter Polytetrafluorethylen(PTFE)-Prothese chirurgisch débridiert wurde.

## Wie lautet Ihre Diagnose?

## Therapieentscheidung

Bei initialem Verdacht auf eine bakterielle Infektion mit Abszessbildung und vorbestehender frischer Implantation eines Prothesenbypasses der ipsilateralen Seite erfolgte das chirurgische Débridement und die Anlage einer Vakuumversieglung, um einer drohenden Protheseninfektion entgegenzuwirken.

### Operativ

Das lokale Débridement erfolgte durch eine 2 × 3 cm Haut-Rand-Exzision am Innenknöchel rechts sowie nach zentral eine Hautlängsinzision. Es entleerte sich altes Hämatom ohne Zeichen auf ein putrides Exsudat. Danach wurde eine ausgiebige Spülung mit Lavanox-Serag^®^ (Wundspüllösung mit < 0,08 % NaOCL/HOCl; Serag Wiesner, Naila, Deutschland), Blutstillung im Situs und Anlage eines „Vacuum-assisted-closure“(V.A.C.)-Verbandes (ActiV.A.C.™ Therapieeinheit, 3M Deutschland GmbH, Neuss, Deutschland) durchgeführt. Das débridierte Gewebe wurde zur histologischen Begutachtung eingesandt.

### Systemisch

**Abb. 3:**
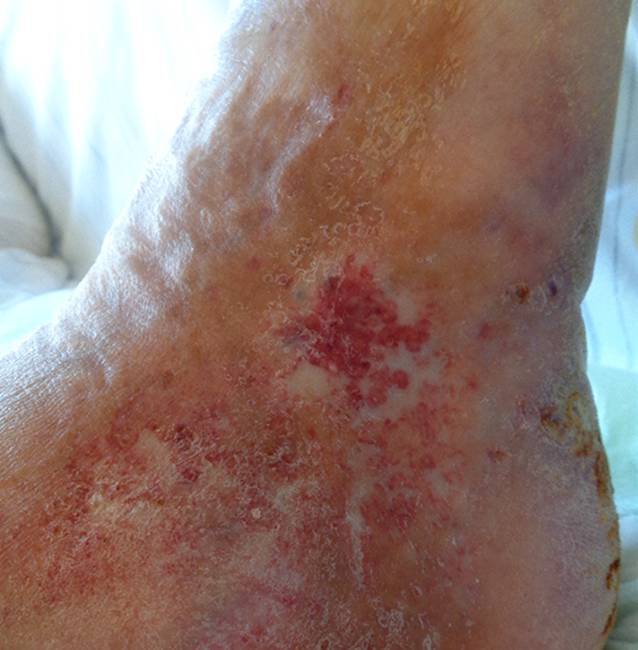
Nachoperative klinische Fotodokumentation des Lokalbefundes am medialen Malleolus der rechten unteren Extremität im stationären Behandlungsverlauf (Quelle: klinischer Bilderfundus – Arbeitsbereich Gefäßchirurgie; Klinik für Allgemein‑, Viszeral‑, Gefäß- und Transplantationschirurgie)

Nach Diagnosestellung des Pyoderma gangraenosum (PG) wurde ein Prednisolonschema mit initial 80 mg, dann Reduktion von 10 mg alle 3 Tage begonnen, unter welchem die bestehenden Ulzera am linken Sprunggelenk unter kleinen Narben abheilten und sich auch am rechten Unterschenkel stabile Wundverhältnisse zeigten. Hier konnte mit adaptierenden Nähten eine Wundverkleinerung erfolgen. Die Prednisolontherapie wurde danach auf eine Erhaltungsdosis von 10 mg/Tag bei Entlassung ausgeschlichen. Unter der systemischen und lokalen Therapie kam es zu einer vollständigen Abheilung der Läsionen (Abb. 3).

## Histopathologie

**Abb. 4:**
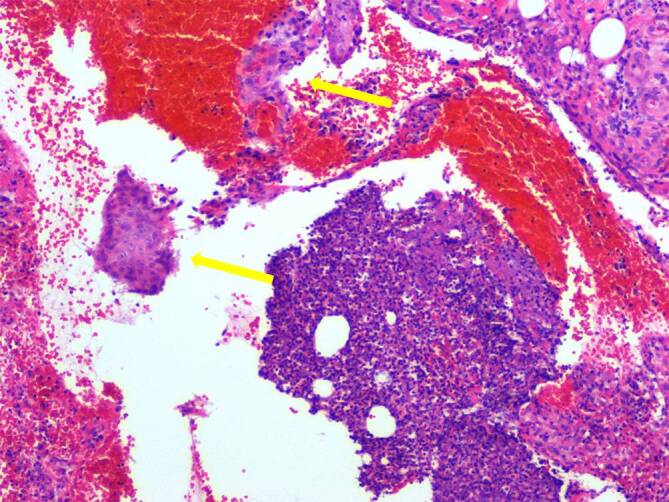
Histopathologische Illustration nekrotischer Exsudate des Lokalbefundes am medialen Malleolus der rechten unteren Extremität, Nekrose mit verwaschenen Zellgrenzen und Gewebsuntergang, kräftig granulozytär durchsetzt und demarkiert und Einschluss winziger Epithelreste (*gelber Pfeil*). (Färbung: Standard Hämatoxylin/Eosin, Vergrößerung.: 100-fach; Quelle: Histopathologischer Bilderfundus – Institut für Pathologie, Klinikum Magdeburg gGmbH, Birkenallee 34, 39130 Magdeburg)

Es kommt Haut‑/Subkutisgewebe zur Darstellung mit Überkleidung durch ein mehrschichtiges Plattenepithel. Dieses reift aus und man sieht eine Akanthose sowie eine Parakeratose. Zentral kommt eine Ulzeration zur Darstellung. In der Tiefe zeigt sich ein dichtes Infiltrat aus Lymphozyten, Plasmazellen und insbesondere neutrophilen Granulozyten mit Übergang in ein abszedierendes Entzündungsbild. Umschrieben dann eine Nekrose mit verwaschenen Zellgrenzen und Gewebsuntergang, kräftig granulozytär durchsetzt, demarkiert und Einschluss winziger Epithelreste. *Zusammenfassung*: Typisches Bild einer PG (Abb. 4).

## Diskussion

### Definition

Das PG zählt zur Gruppe der neutrophilen Dermatosen. Diese Gruppe von Hautkrankheiten zeichnet sich histologisch durch kutane aseptische Abszesse und neutrophile Infiltrate aus. Zu den neutrophilen Dermatosen gehören unter anderem auch das Sweet-Syndrom, der Morbus Behçet und die generalisierte Psoriasis pustulosa [1]. Eine entscheidende Rolle scheint eine genetische Prädisposition zu spielen. So könnte das pathogene Gen des Tyrosin-Protein-Phosphatase-Non-Receptor Typ 6 (PTPN6) und die verminderte Aktivität seines Proteins sowie die pathogene Variante des Prolin-Serin-Threonin-Phosphatase-interagierenden-Protein-1-Gens (PSTPIP1) zur Aktivierung eines Inflammasoms führen, was seinerseits Interleukin 1β (IL-1β) aktiviert, das indirekt Neutrophile rekrutiert und einen autoinflammatorischen Zustand verursacht. PG kann durch ein Trauma ausgelöst oder verschlimmert werden (Pathergiephänomen). Physische Verletzungen können die Freisetzung von IL-36 und Autoantigenen aus geschädigten Keratinozyten verursachen. So sind IL-36A und IL-36G, die von Epithelzellen produziert werden, für ihre Rolle bei vielen Autoimmunerkrankungen wie Psoriasis, Hidradenitis suppurativa, akuter generalisierter Pustulose, Morbus Crohn oder Colitis ulcerosa bekannt. So ist anzunehmen, dass ein Trauma mit Freisetzung von RNA durch Keratinozyten zur Aktivierung der angeborenen Immunantwort und zur Degranulation von Neutrophilen sowie zur Freisetzung von IL-36 und zur Aktivierung dieses Zytokins durch IL-36-abgeleitete Neutrophilenproteasen führt. IL-36 hat eine entzündungsfördernde (proinflammatorische) Wirkung, indem es die Neutrophilen zur verstärkten Expression entzündungsfördernder Zytokine anregt und die Differenzierung naiver T‑Zellen in Richtung der Th1-Linie fördert [2].

**Diagnose:** Pyoderma gangraenosum (PG) nach Revaskularisation des rechten Beines bei inkompletter Ischämie mit einem femoroinfragenualen Prothesenbypass bei begleitender Colitis ulcerosa

Die häufigste Grunderkrankung beim Auftreten einer PG ist eine chronisch-entzündliche Darmerkrankung. Kridin et al. konnten in ihrer Metaanalyse zeigen, dass die Prävalenz einer Colitis ulcerosa in den verschiedenen Studienpopulationen zwischen 3,4 und 32,3 % lag, während die kombinierte Prävalenz mit 11,5 (95 %-CI: 7,2–16,6) % berechnet wurde [3].

### Diagnostik

Die Diagnose des PG erfolgt in der Regel klinisch, d. h. anhand der Symptome und des klinischen Erscheinungsbildes der Geschwüre. Dieses Vorgehen ist allerdings sehr von der Erfahrung der Therapeuten abhängig und fehleranfällig [4]. Der entwickelte PARACELSUS-Score fasst verschiedene Diagnosekategorien in Haupt‑, Neben- und Zusatzkriterien zusammen. Labor- oder Blutuntersuchungen sind für die Diagnosestellung nicht erforderlich.

### Therapieoptionen

**Abb. 5:**
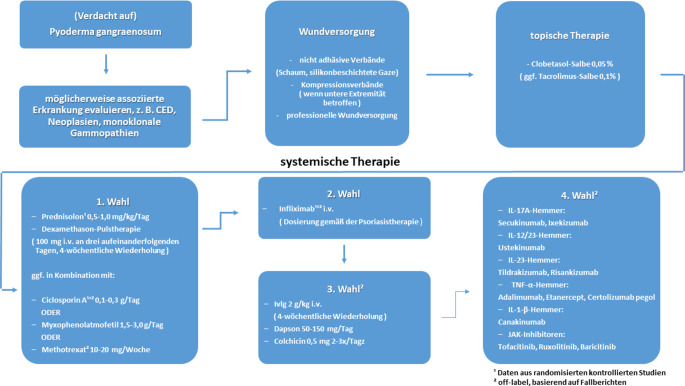
Algorithmus zur Therapie des Pyoderma gangraenosum. (Aus [1])

Die Behandlung des PG ist multimodal und umfasst modernes Wundmanagement, Schmerztherapie, topische sowie systemische immunsuppressive bzw. -modulatorische Therapeutika (Abb. 5; [1]), die jeweils an den Schweregrad und den Verlauf der Erkrankung anzupassen sind. Daher kommt es im Wesentlichen darauf an, die Erkrankung frühzeitig zu erkennen, um Fehleinschätzungen und eine unnötige operative Therapie zu vermeiden. Das lokale Débridement mit Anlage einer Vakuumversieglung unter dem Verdacht einer bakteriellen Infektion stellte – selbstkritisch gesehen – nicht die wirklich voll befundgerechte Therapiemaßnahme dar und führte im Sinne des o. g. Pathomechanismus wahrscheinlich eher zu einer Verschlechterung der Situation. Unter dem Eindruck der schnellen Regression im Zuge der Prednisolontherapie verstärkt sich der Eindruck der Fehleinschätzung, jedoch überwog initial die Sorge vor einer Infektion des gleichseitig frisch angelegten Prothesenbypasses. Des Weiteren hätte der akute Schub der bekannten Colitis ulcerosa im Zusammenhang mit dem frischen vorliegenden Trauma an das PG denken lassen müssen. Dies unterstreicht, dass das PG nach wie vor eine diagnostische Herausforderung darstellt, da es sich um eine seltene Erkrankung mit mehreren klinischen Nachahmern handelt. Mit einer Fehldiagnoserate von bis zu 39 % gilt es als Prototyp der Fehldiagnose unter den Hautgeschwüren, bei denen die Erstdiagnose PG war. Die häufigsten klinischen Nachahmer sind venöse Beingeschwüre, Vaskulitis, Vaskulopathien und faktitiale Ulzera [5].

## Fazit für die Praxis

Das PG stellte eine Herausforderung im klinischen Alltag dar, da das therapeutische Vorgehen wesentlich von der schwierig zu stellenden Diagnose abhängt. Die Fehldiagnoserate ist mit 39 % sehr hoch. Insbesondere das Vorhandensein einer chronisch-entzündlichen Darmerkrankung im Zusammenhang mit einem frischen operativen Trauma sollte den Chirurgen beim Auftreten der beschriebenen pustulösen Effloreszenzen an eine PG denken lassen. Ein chirurgisches Débridement der Läsionen sollte vermieden werden. Die Behandlung ist multimodal und beinhaltet ein modernes Wundmanagement, eine adäquate Schmerztherapie, topische sowie systemische immunsuppressive bzw. immunmodulatorische Therapeutika.
